# Radionuclides proportion and radiological risk assessment of soil samples collected in Covenant University, Ota, Ogun State Nigeria

**DOI:** 10.1016/j.mex.2018.10.023

**Published:** 2018-10-29

**Authors:** O.O. Adewoyin, M. Omeje, E.S. Joel, S.A. Akinwumi, C.O. Ehi-Eromoseled, Zaidi Embong

**Affiliations:** a Department of Physics, Covenant University, Ota, Ogun State, Nigeria; b Department of Chemistry, Covenant University, Ota, Ogun State, Nigeria; c Department of Physics, Universiti Tun Hussein Onn Malaysia

**Keywords:** Covenant University, Gamma-ray, Activity concentrations, Radiological hazards

## Abstract

The activity levels of ^238^U, ^232^Th and ^40^K in soil surrounding major office complexes in Covenant University were analyzed for radiological hazards to determine the safety of the residents in such environment. Sixteen (16) soil samples were collected, prepared and sent to Acme laboratory in Canada for analysis with the use of high purity germanium detector. The mean activity concentrations of ^238^U, ^232^Th and ^40^K were found to be 45 ± 10, 135 ± 8 and 195 ± 20 respectively. The concentrations of ^238^U and ^232^Th were found to be higher than the world recommended standard of 35 and 30, while the result for ^40^K was noted to be lower than the world safe limit. The average values of Raeq, D, AED, I_yr_, H_ex_ and ELCR in this study were estimated to be 252.33 Bq/kg, 110.15 nGy/h, 0.13 mSv/y, 1.78, 0.68 and 0.47 × 10^−3^ respectively. It was noticed that none of the measured parameters was higher than the internationally recommended safe limits. ^232^Th was found to be the major contributor to the environmental radionuclides in the area of study. Therefore, the inhabitants of the office complexes whose environment was assessed are considered not be exposed to any radiological hazards.

## Graphical abstract

Survey plan for the Study.

**Figure:**
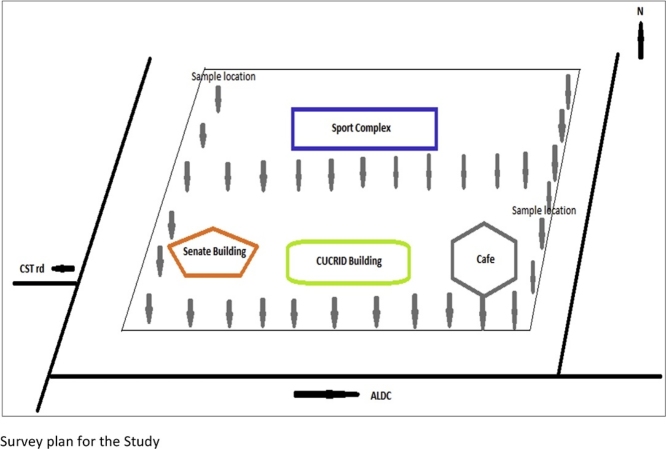

## Introduction

Living things are consistently and continuously exposed to background ionizing radiation, whose origin could be both natural and man-made [1]. Natural radionuclides spread widely in soils, sediments and rocks. Natural background radiation is determined by the activity of the radionuclide content in soil [2,3]. Although, geologic features as well as the activities of man can trigger the level of radioactivity in soil [4,5]. For instance, rocks that are rich in granite, phosphate and salt are often rich in natural radionuclides, which find their ways into the soils in the environment by weathering and other mechanical transportation processes. Similarly, human activities such as industrialization, agricultural practices, mining, building materials and so on can influence the radioactivity level in soil.

Previous studies have shown that man is exposed to natural background radiation in the region of 2.4 mSv, which accounts for about 80 percent of the annual radiation dose exposure per person per annum [6,7]. That being said, building is an essential part of man’s life in the modern world because man lives, works, schools and does his activities in one building or another [30]. Thus, adequate knowledge of the natural radioactivity in soil will assist in evaluating the degree of radiation exposure to the population and it will also help in determining the appropriate locations to site residential and office structures.

The radiological consequences of undue exposure to these radionuclides to man can lead to major hazard effect such as the irradiation of the lung tissue and cancer [8]. In order to avoid these kinds of disease, it is essential to assess the natural environmental radioactivity and its associated gamma radiation so as to ascertain the safety of the residents of the geographical location. Dizman et al. [3] established the possibility of the annual effective gamma doses and life time cancer risks being higher than the world’s recommended average, while investigating the background radiation level in soil in order to estimate the associated health risks. Korkmaz et al. [7] observed that adequate information on the distribution of radionuclide in the environment is very expedient for radiation measurement and protection [9,10]. Furthermore, Kumari et al. [8] is of the opinion that the effects of radiation exposure to man can be estimated by evaluating the levels of radioactive pollutants that is emitted to the environment [28,29].

It is on this note that this study was conducted using High Purity Germanium detector to determine the concentration of naturally occurring radionuclides and other radiological parameters as well as assessing the contribution of each radionuclide to these radiological parameters, in the soil samples collected in the areas surrounding Covenant University Centre for Research Innovation and Development (CUCRID), Senate building, Sports Complex and Café 1 all in Covenant University, Ota, Ogun State, in order to evaluate the health risk, the population of about 10,000 people that have their activities within this area on a daily basis are exposed to.

### Study area

**Fig. 1:**
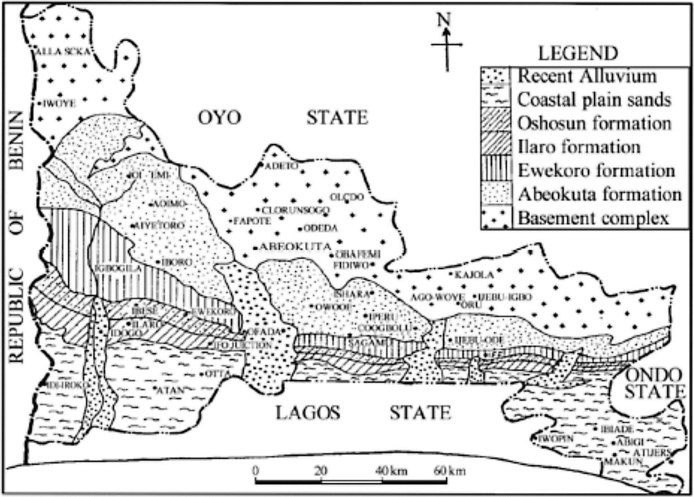
Geological map of the study area [4].

The study was conducted in Covenant University around the CUCRID building, Senate building, Sports Complex and Café area. The area of study is located within latitude 6° 40′ 26.17″ to 6° 40′19.38″ N and longitude 3° 09′ 37.09″ to 3° 09′ 44.57″ E of the Dahomey basin. The geology of this area has been largely discussed by many authors in which the stratigraphy has been grouped into six lithostratigraphic formations namely from the ancient to the youngest as Abeokuta, Ewekoro, Akinbo, Oshosun, Ilaro and Benin formations. Abeokuta formations is composed of Ise, Afowo and Araromi formations (Fig. 1).

## Materials and methods

### Sample collection and preparation

**Fig. 2:**
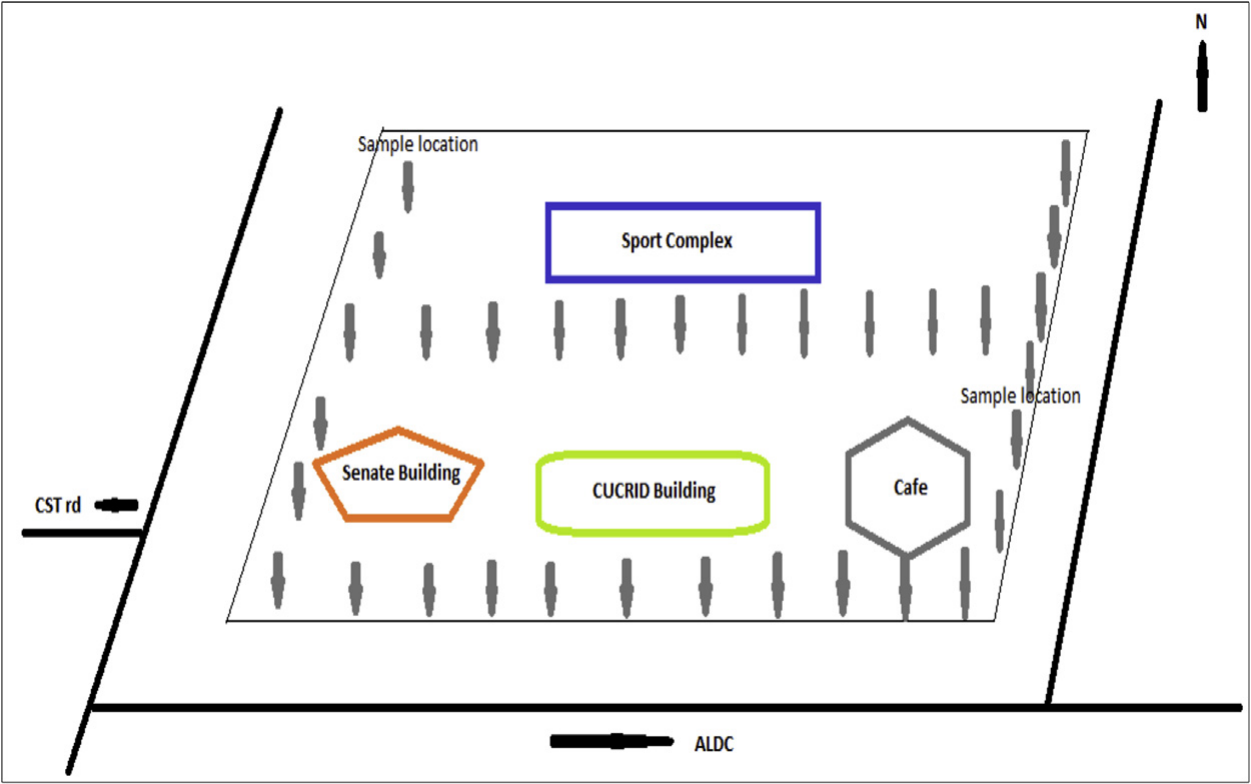
Base map of the study area and sample locations.

Soil samples were collected around CUCRID building, Senate Building, Sports Complex and Café 1 building (Fig. 2). Four samples were collected at every sampling location at a depth of about 0–15 cm in the listed areas. A square area (1 m x 1 m) was measured and sample was collected in each of the diagonals and 16 m was the interval between each sampling location. Sixteen (16) samples were collected around the region considered for this study and packaged in plastic bags. Initial labelling of the samples was done for easy identification and separation. All samples were transported to the Covenant University Chemistry Research Laboratory, where each sample was oven dried at a temperature of 105–110 °C. The dried samples were crushed using mortar and pestle to produce fine particle size and the particle was sieved using 75 μm mesh size in order to remove dried leaves and stone pebbles. Finally, 60 gm was measured out of each sample using a weigh balance and poured in plastic bags that are labelled according to their original container. The prepared samples were packaged and sent to Activation Laboratories Limited in Canada for analysis.

### Analysis of the sample

The samples were weighed in 2 Oz seamless tin canisters (2⅙” diameter x 1⅜” height) and sealed with an electrical tape for four weeks to achieve secular equilibrium between parent’s uranium and thorium with their daughter radionuclides. The high-resolution Gamma spectroscopy spectrums are acquired after 10000 s count. The ^226^Ra (^238^U) and ^232^Th are determined from their progenies (^214^Pb & ^214^Bi for ^226^Ra and ^212^Pb & ^228^Ac for ^232^Th) [11,12].

### Quality assurance and calibration standards

The Gamma spectrometry system is calibrated with Certified Reference Standards by CANMET (DL-1a, BL-4a, BL-5, DH-1a and UTS-2), USGS (STM-2), IAEA – 372 and IAEA - 447. QA includes blank, duplicate and control standards (DL-1a and UTS-2) in every batch of 20 samples. The counting geometry is identical for all samples and standards [13,14].

### Estimation of radiological parameters

These parameters were estimated in order to assess the level of exposure of the residents of the study area to background ionizing radiation.

The activity concentrations of ^238^U (^226^Ra), ^232^Th and ^40^K were measured in each of the soil samples.

Radium equivalent activity is a popularly assessed hazard index and it is evaluated with the use of the proposed by Beretka and Mathew [15] in Eq. (1). In this relation, it is assumed that 370 Bq/kg of ^226^Ra, 259 Bq/kg of ^232^Th and 4810 Bq/kg of ^40^K produce the same gamma dose rate

(1) $${Ra}_{eq}=A_{Ra}+1.43A_{Th}+0.077A_{K}$$

Where A_Ra_, A_Th_ and A_K_ are the activity concentrations of ^226^Ra, ^232^Th and ^40^K in Bq/kg, respectively [16,17].

Another characteristic of the external terrestrial gamma radiation, the absorbed dose rate (D) in air at 1 m above the ground surface owing to the concentration of radionuclides was calculated by Eq. (2) according to OECD [18] and UNSCEAR [19].

(2) $$\text{D}\;(\text{n}\text{G}\text{y}/\text{h})=0.462C_{U}+0.604C_{Th}+0.0417C_{K}$$

Where C_U_, C_Th_ and C_K_ are the activity concentrations in Bq/kg of ^238^U, ^232^Th and ^40^K in soil samples, respectively. 0.462, 0.604 and 0.0417 are the conversion factors for uranium, thorium and potassium, respectively in the samples.

To estimate the annual effective dose, consideration must be given to the conversion coefficient from absorbed dose in air to effective dose and the outdoor occupancy factor. The annual estimated average effective dose equivalent received by a member is calculated using a conversion factor of 0.7 Sv/Gy, which is used to convert the absorbed dose rate to annual effective dose with an outdoor occupancy of 20% [20,21].

The annual effective dose was determined using Eq. (3)

(3) AED = D (nGy/h) × 8760 h × 0.2 × 0.7 SvG/y × 10^−6^

Where D is the absorbed dose rate in air (nGy/ h), 0.7 is the dose conversion factor, 0.2 is the outdoor occupancy factor while 8760 h/y is the time [[22], [23], [24]].

One other radiation hazard index is called the representative level index, Iγr, and it is defined by Eq. (4) as

(4) $$I_{yr}=\frac{1}{150Bq/kg}A_{Ra}+\frac{1}{100Bq/kg}A_{Th}+\frac{1}{1500Bq/kg}A_{K}$$

Where ARa, ATh, and AK have the same meaning as in Eq. (1).

The external hazard index, Hex was calculated for the investigated samples using the model proposed by Raghu et al. [25] and Joel et al. [12] where the external hazard index is given by Eq. (5)

(5) $$H_{ex\;}=\;\frac{A_{Ra}}{370}+\frac{A_{Th}}{259}+\frac{A_{K}}{4810}\;\leq 1$$

Excess life time cancer risk (ELCR) was calculated using Eq. (6)

(6) ELCR = AEDE x DL x RF

Where DL is the duration of life (70 years) and RF is risk factor (S/v). For stochastic effects, ICRP 90 uses values of 0.05 for the public [5,26].

## Results and discussion

The results of the measured activity concentrations from the soil samples collected from sixteen (16) locations are presented in Table 1. It could be observed that the activity concentration of ^238^U (^226^Ra) ranged between (32 ± 8) and (78 ± 14) Bq/kg. The minimum value was noticed in Senate 6 while the maximum value was observed in Sport 17. Similarly, from the activity concentrations of the soil samples, it was noted from the result that ^232^Th varied from (78 ± 6) in Sport 22 to (483 ± 14) Bq/kg in Sport 17. Furthermore, the concentrations of measured in the samples for ^40^K was between senate 2 and Sport 17 with values ranging between (66 ± 10) and (923 ± 39) Bq/kg respectively. The activity concentration of ^40^K was noticed to be higher than ^238^U and ^232^Th in all the measured samples. The estimated average values for ^238^U, ^232^Th and ^40^K are (45 ± 10), (135 ± 8) and (145 ± 20) Bq/kg respectively. In addition, the average activity concentrations for ^238^U and ^232^Th in the present study, were found to be higher than the world recommended standard of 35 and 30 Bq/kg, while it is lower in ^40^K than the world standard of 400 Bq/kg as reported by UNSCEAR [19]. The result of the activity concentrations of ^238^ U and ^232^Th in the present study are higher than the result obtained in Dizman et al. [3] and Usikalu et al. (2017), while the activity level of ^40^K, in the current research, is far lower than the result of the previous study. Furthermore, the activity concentration of 238U in Amanjeet et al. [27] corresponds with the activity levels of ^238^U in the present study while the concentration levels of ^232^Th and ^40^K in the previous study were far higher than the present study. The variations observed in the results of the present study could be as a result of the differences in the local geology and mineralogical composition of the study area when compared to other regional geology.

**Table 1:** Radioactivity Concentrations of ^238^U, ^232^Th and ^40^K in the Soil samples.

s/n	Sample Identity	^238^U (Bq/kg)	^232^Th (Bq/kg)	^40^K (Bq/kg)
1	CUCRID 7	40 ± 10	127 ± 9	131 ± 18
2	CUCRID 8	46 ± 10	144 ± 9	197 ± 21
3	CUCRID 13	41 ± 9	123 ± 8	145 ± 19
4	CUCRID 27	49 ± 10	119 ± 9	142 ± 21
5	SENATE 2	47 ± 10	112 ± 9	66 ± 10
6	SENATE 5	35 ± 9	82 ± 6	175 ± 17
7	SENATE 6	32 ± 8	97 ± 6	144 ± 15
8	SENATE 4	47 ± 10	88 ± 6	102 ± 17
9	SPORT 17	78 ± 14	483 ± 14	923 ± 39
10	SPORT 8	47 ± 10	110 ± 8	154 ± 19
11	SPORT 22	35 ± 8	78 ± 6	130 ± 16
12	SPORT 12	37 ± 10	156 ± 9	254 ± 24
13	CAFÉ 4	48 ± 9	118 ± 8	127 ± 20
14	CAFÉ 3	46 ± 11	119 ± 9	167 ± 21
15	CAFÉ 6	42 ± 10	85 ± 6	132 ± 17
16	CAFÉ 9	50 ± 10	111 ± 8	127 ± 20
	**mean**	45 ± 10	135 ± 8	195 ± 20

The results of the radium equivalent activity Ra_eq_ in the present study ranged between 156.55 at Sport 22 and 839.76 Bq/kg at Sport 17 with a mean value of 252.33 Bq/kg. This result is below the permissible limit of 370 Bq/kg recommended by OECD [18]. The absorbed dose rate in the soil samples in the area of study varied from 68.7 nGy/h (Sport 22) to 366.26 nGy/h (Sport 17) with an average value of 110.15 nGy/h. The air absorbed dose was noticed to be higher than population weighted average value of 59 nGy/h for primordial radiation [19]. The annual effective dose was estimated to quantify the radiological risk of radionuclides in soil to the inhabitants and the results are presented in Table 2. The annual effective dose (AED) ranged between 0.08 and 0.45 mSv/y with a mean value of 0.13 mSv/y. The minimum value of AED was noticed at Sport 22, while the maximum result was obtained at Sport 17. The average value of the AED was observed to be higher than the world recommended safe limit of 0.07 mSv/y by an approximate factor of 2. The result of AED in the present study agreed with Zhang [1], while it is much lower than what was obtained in Amanjeet et al. [27].

**Table 2:** Radiological Parameters considered in the Present Study.

s/n	Sample ID	Ra_eq_ (Bq/kg)	D (nGy/h)	AED (mSv/y)	Iyr	Hex	ELCR (x 10^−3^)
1	CUCRID 7	231.7	100.65	0.12	1.63	0.63	0.42
2	CUCRID 8	267.09	116.44	0.14	1.88	0.72	0.49
3	CUCRID 13	228.06	99.28	0.12	1.60	0.61	0.42
4	CUCRID 27	230.1	100.44	0.12	1.62	0.62	0.42
5	SENATE 2	212.24	92.11	0.11	1.47	0.57	0.39
6	SENATE 5	165.74	73.0	0.09	1.17	0.45	0.32
7	SENATE 6	181.8	79.37	0.10	1.28	0.49	0.35
8	SENATE 4	180.69	79.11	0.10	1.26	0.49	0.35
9	SPORT 17	839.76	366.26	0.45	5.97	2.26	1.60
10	SPORT 8	216.16	94.57	0.12	1.51	0.58	0.42
11	SPORT 22	156.55	68.7	0.08	1.10	0.42	0.28
12	SPORT 12	279.64	121.9	0.15	1.98	0.75	0.53
13	CAFÉ 4	226.52	98.75	0.12	1.59	0.62	0.42
14	CAFÉ 3	229.03	100.09	0.12	1.61	0.61	0.42
15	CAFÉ 6	173.71	76.24	0.09	1.22	0.47	0.32
16	CAFÉ 9	218.51	95.44	0.12	1.53	0.60	0.42
**Average**		**252.33**	**110.15**	**0.13**	**1.78**	**0.68**	**0.47**

**Fig. 3:**
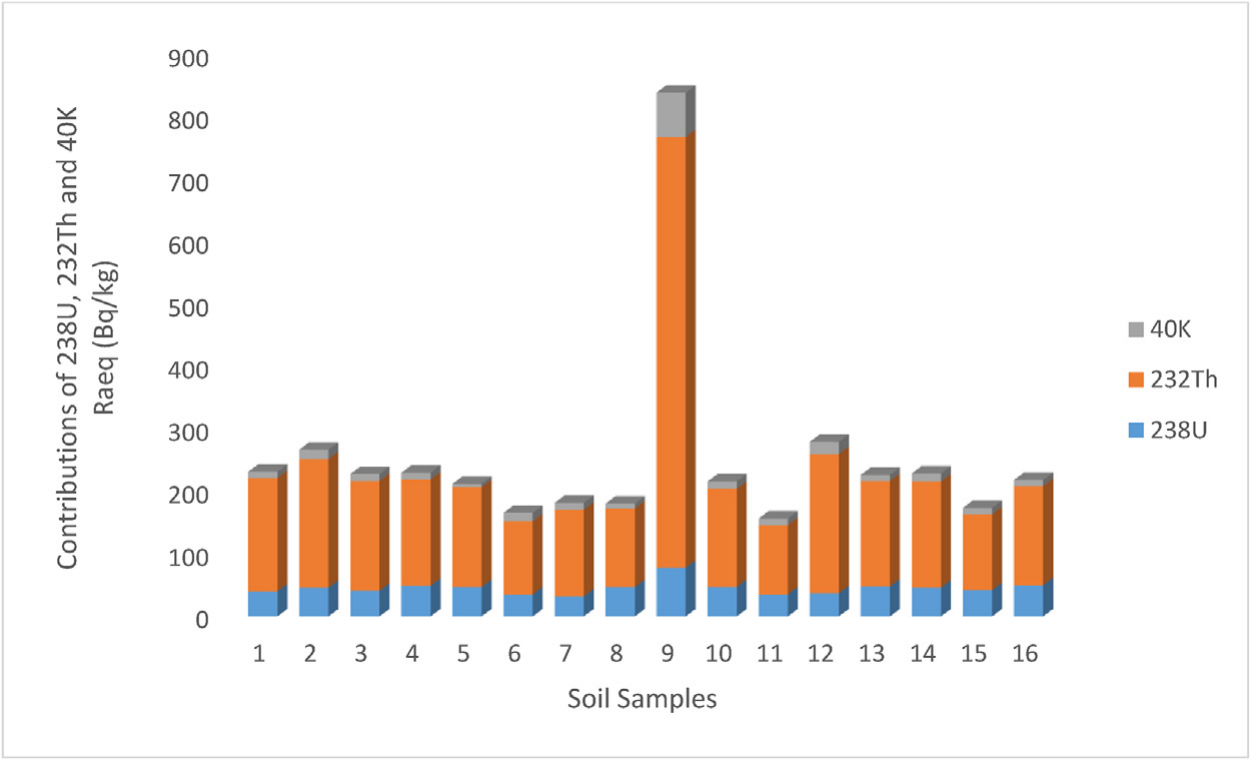
The contributions of ^238^U, ^232^Th and ^40^K to Raeq in each Sample.

**Fig. 4:**
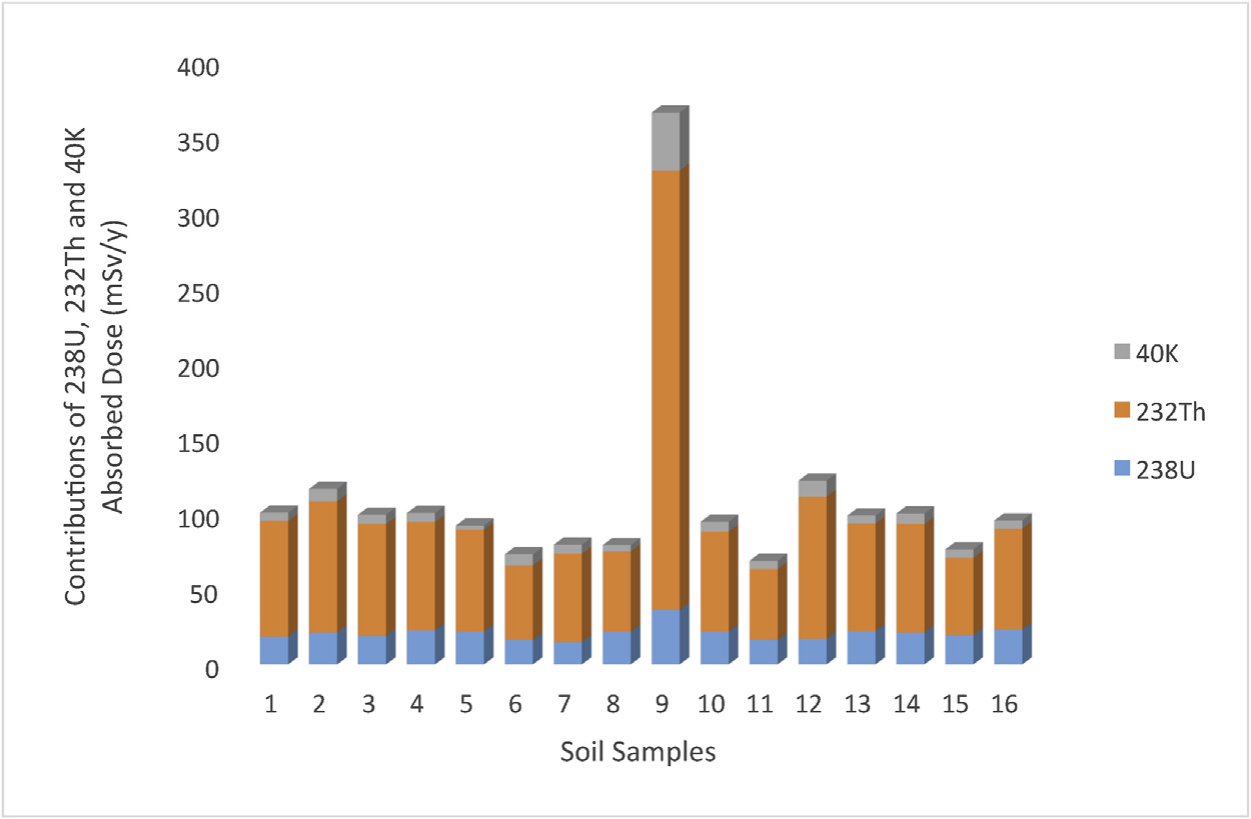
The contributions of ^238^U, ^232^Th and ^40^K to D (mSv/y) in each Sample.

In addition, the result of the representative level index I_yr_, varied from 1.10 (Sport 22) to 5.97 (Sport 17), with a mean value of 1.78. The obtain result agreed with Dizman et al. [3] but lower than the result in Kumari et al. [8]. The results of I_yr_ in the present study correlated with the recommended standard of I_yr_ ≤ 2 as a result, the soil samples could be said not to pose any significant risk to the inhabitants of the area of study. The results of the external hazard index (Hex) ranged between 0.42 and 2.26 with a mean value of 0.68. Although, the value of Hex at Sport 17 is far higher than the recommended standard of unity but a sample point in a collection of sample points may not be sufficient to expose residents to radiological hazard emanating from the environment. The result of H_ex_ in the present study agreed with Korkmaz et al. [7] but higher than the results of Amanjeet et al. [27]. The results of ELCR for the studied soil samples ranged between 0.28 × 10^−3^ and 1.60 × 10^−3^ with an average value of 0.47 × 10^−3^. The mean result of ELCR do not exceed the recommended safe limit of 3.75 × 10^-3^ [5]. The result of ELCR in the present study is comparable to the result obtained at the Rize province in Turkey [3]. The variations noticed in the radiological parameters of the soil samples at different locations of the study area could be as a result of the variation in the mineralogical composition and the geological formation of the study area. It was also observed in all the estimated radiological parameters that ^232^Th had the highest contribution to the environmental radioactivity in all the sample locations (Fig. 3, Fig. 4). The contributions of Thorium in all the radiological parameters varied between 70 and 85 percent.

## Conclusion

High purity germanium detector was used to analyze soil samples around the major office complexes in Covenant University in order to determine effects of radiation from the environment on the inhabitant of the offices. The activity concentrations of ^238^U and ^232^Th were found to be higher than the world permissible level on the other hand, the activity concentration of ^40^K was lower than the world standard. Furthermore, the radiological parameters estimated such as the Raeq, D, AED, I_yr_, H_ex_ and ELCR were found to be within the recommended safe standards. Moreover, ^232^Th was observed to be the major contributor to the environmental radionuclides in the area of study. Thus, the occupants of the office spaces are not in any risk of exposure to any radiological risk from their immediate environment.
